# Evidence for national universal eye health plans

**DOI:** 10.2471/BLT.18.213686

**Published:** 2018-08-27

**Authors:** Jacqueline Ramke, Anthony B Zwi, Juan Carlos Silva, Nyawira Mwangi, Hillary Rono, Michael Gichangi, Muhammad Babar Qureshi, Clare E Gilbert

**Affiliations:** a Faculty of Infectious & Tropical Diseases, London School of Hygiene & Tropical Medicine, Keppel Street, London WC1E 7HT, England.; b Health, Rights and Development, School of Social Sciences, University of New South Wales, Sydney, Australia.; c World Health Organization, Bogotá, Colombia.; d Department of Clinical Medicine, Kenya Medical Training College, Nairobi, Kenya.; e Department of Ophthalmology, Kitale County and Referral Hospital, Kitale, Kenya.; f Ophthalmic Services Unit, Ministry of Health, Nairobi, Kenya.; g Christian Blind Mission, Cambridge, England.

## Abstract

Many low- and middle-income countries use national eye-care plans to guide efforts to strengthen eye-care services. The World Health Organization recognizes that evidence is essential to inform these plans. We assessed how evidence was incorporated in a sample of 28 national eye-care plans generated since the *Universal eye health: a global action plan 2014–2019* was endorsed by the World Health Assembly in 2013. Most countries (26, 93%) cited estimates of the prevalence of blindness and 18 countries (64%) had set targets for the cataract surgical rate in their plan. Other evidence was rarely cited or used to set measurable targets. No country cited evidence from systematic reviews or solution-based research. This limited use of evidence reflects its low availability, but also highlights incomplete use of existing evidence. For example, despite sex-disaggregated data and cataract surgical coverage being available from surveys in 20 countries (71%), these data were reported in the eye health plans of only nine countries (32%). Only three countries established sex-disaggregated indicators and only one country had set a target for cataract surgical coverage for future monitoring. Countries almost universally recognized the need to strengthen health information systems and almost one-third planned to undertake operational or intervention research. Realistic strategies need to be identified and supported to translate these intentions into action. To gain insights into how a country can strengthen its evidence-informed approach to eye-care planning, we reflect on the process underway to develop Kenya’s seventh national plan (2019–2023).

## Résumé

De nombreux pays à revenu faible et intermédiaire ont recours à des plans nationaux de santé oculaire pour guider les actions visant à renforcer les services d'ophtalmologie. L'Organisation mondiale de la Santé reconnaît qu'il est essentiel de disposer de données factuelles pour orienter ces plans. Nous avons évalué la manière dont ces données factuelles ont été intégrées à un échantillon de 28 plans nationaux de santé oculaire, élaborés depuis l'adoption par l'Assemblée Mondiale de la Santé, en 2013, du document *Santé oculaire universelle: plan d'action mondial 2014*–*2019*. La plupart des pays (26, soit 93%) ont indiqué utiliser des estimations de la prévalence de la cécité et 18 pays (64%) avaient fixé des objectifs relatifs au taux de chirurgie de la cataracte dans leur plan. D'autres types de données factuelles ont rarement été mentionnés ou utilisés pour définir des objectifs mesurables. Aucun pays n'a mentionné de données issues de revues systématiques ou de recherches fondées sur des solutions. Cette utilisation limitée des données factuelles reflète leur faible accessibilité, mais aussi l'usage incomplet des données existantes. Par exemple, bien que des enquêtes menées dans 20 pays (71%) donnent accès à des données ventilées par sexe et au taux de couverture de la chirurgie de la cataracte, seuls neuf pays (32%) ont reporté ces données dans leur plan de santé oculaire. Seuls trois pays ont mis en place des indicateurs ventilés par sexe et un seul a défini un objectif de couverture de la chirurgie de la cataracte pour en suivre l'évolution. La quasi-totalité des pays a reconnu qu'il était nécessaire de renforcer les systèmes d'information sanitaire et près d'un tiers prévoyait d'entreprendre des recherches opérationnelles ou interventionnelles. Il faudra définir et mettre en œuvre des stratégies réalistes pour passer de l'intention à l'action. Pour en savoir plus sur la manière dont un pays peut renforcer son approche d'élaboration de plans de santé oculaire à partir de données factuelles, nous nous intéressons à l'élaboration, en cours, du septième plan national du Kenya (2019–2023).

## Resumen

Muchos países con ingresos entre bajos y medios utilizan planes nacionales de atención oftalmológica para orientar los esfuerzos a fortalecer los servicios de atención oftalmológica. La Organización Mundial de la Salud reconoce que las pruebas son esenciales para informar a estos planes. Se evaluó cómo se incorporaron las pruebas en una muestra de 28 planes nacionales de atención oftalmológica generados desde que la Asamblea Mundial de la Salud aprobó *Universal eye health: a global action plan 2014–2019* (Atención oftalmológica universal: un plan de acción mundial para 2014-2019) en 2013. La mayoría de los países (26, 93 %) citaron estimaciones de la prevalencia de la ceguera y 18 países (64 %) habían establecido metas para la tasa quirúrgica de cataratas en sus planes. Rara vez se citaron o utilizaron otras pruebas para establecer objetivos mensurables. Ningún país citó pruebas de revisiones sistemáticas o investigaciones basadas en soluciones. Este uso limitado de las pruebas refleja su baja disponibilidad, pero también destaca el uso incompleto de las pruebas existentes. Por ejemplo, a pesar de que los datos desglosados por sexo y la cobertura quirúrgica de cataratas están disponibles en las encuestas de 20 países (71 %), estos datos solo se reflejaron en los planes de atención oftalmológica de nueve países (32 %). Solo tres países establecieron indicadores desglosados por sexo y solo un país había establecido una meta para la cobertura quirúrgica de cataratas para el seguimiento futuro. Los países reconocieron casi universalmente la necesidad de fortalecer los sistemas de información sanitaria y casi un tercio tenía previsto realizar investigaciones operacionales o de intervención. Es necesario identificar y apoyar estrategias realistas para convertir estas intenciones en acciones. Para comprender mejor cómo un país puede fortalecer su enfoque basado en pruebas para la planificación de la atención oftalmológica, se ha analizado el proceso en curso para desarrollar el séptimo plan nacional en Kenia (2019-2023).

## ملخص

تلجأ العديد من البلدان ذات الدخل المنخفض والمتوسط إلى البرامج الوطنية لرعاية العيون بهدف توجيه الجهود لتعزيز خدمات رعاية العيون. وتدرك منظمة الصحة العالمية أن هذا الدليل ضروري بهدف إثراء هذه البرامج. وقمنا بتقييم كيفية دمج الأدلة في عينة من 28 برنامجاً وطنياً لرعاية العيون تم إنشاؤها بعد التصديق على خطة *الصحة الشاملة للعين: وهي خطة عمل للفترة 2014 إلى 2019، *تم اعتمادها بواسطة جمعية الصحة العالمية في عام 2013. أشارت معظم البلدان (26 دولة، بنسبة 93٪) إلى تقديرات حول انتشار العمى، وحددت 18 بلدا ( بنسبة 64٪) أهدافاً لمعدل جراحة الكتاراكت (إعتام العدسة) في برامجها. ونادرا ما تم الإشارة إلى أدلة أخرى أو استخدامها لوضع أهداف قابلة للقياس. لم يذكر أي بلد أدلة من المراجعات المنهجية أو البحث القائم على الحلول. إن هذا الاستخدام المحدود للأدلة يعكس توافرها المحدود، ولكنه يسلط الضوء أيضًا على الاستخدام غير الكامل للأدلة الموجودة. فمثلاً، على الرغم من توافر البيانات غير المصنفة حسب الجنس، وكذلك توافر التغطية الجراحية للكتاراكت (إعتام العدسة) في المسوحات في 20 بلداً (71٪)، فقد تم الإبلاغ عن هذه البيانات في برامج صحة العيون في تسعة بلدان فقط (32٪). وأنشأت ثلاثة بلدان فقط مؤشرات غير مصنفة حسب نوع الجنس، وحدد بلداً واحداً فقط هدفاً للتغطية الجراحية للكتاراكت (إعتام العدسة) من أجل الرصد المستقبلي. لقد أدركت البلدان تقريباً الحاجة إلى تقوية أنظمة المعلومات الصحية، وخطط ثلثها تقريبًا للاضطلاع بأبحاث تشغيلية أو تدخلية. يجب تحديد استراتيجيات واقعية ودعمها لترجمة هذه النوايا إلى عمل. للحصول على معلومات حول كيفية تعزيز بلد ما لمنهجه القائم على الأدلة في التخطيط للعناية بالعين، فإننا ندرس العملية الجارية لتطوير البرنامج الوطني السابع لكينيا (2019 إلى 2023).

## 摘要

许多低收入和中等收入国家采用国家普遍眼健康计划来指导加强眼保健服务。世卫组织认识到，在推行这些计划的过程中，证据十分必要。自 2013 年世界卫生大会通过《普遍的眼健康——2014-2019 年全球行动计划》以来，28 个国家出台了全国性的眼保健计划，我们评估了证据是如何被纳入此样本的。大多数国家（26 个国家，占 93％）引用了盲症患病率估值，18 个国家（占 64%）在计划中设定了白内障手术率目标。很少引用或使用其他证据以设定可衡量目标。没有国家引用系统评价证据或基于解决方案的研究证据。证据的有限使用反映了其低可用性，但也突出了现有证据的不完全使用。例如，尽管 20 个国家（占 71%）的调查显示了按性别分列的数据和白内障手术覆盖范围，但这些数据仅在 9 个国家（占 32%）的眼保健计划中有所报告。只有 3 个国家制定了按性别分列的指标，1 个国家为未来的监测设定了白内障手术覆盖范围的目标。各国几乎普遍认识到加强卫生信息系统的需要，近三分之一的国家计划开展操作性或干预性研究。需要确定并支持切合实际的战略，将意愿转化为行动。为深入了解各国如何强化其眼保健计划的循证方法，我们反思了正在进行中的进程以制定肯尼亚的第七个国家计划 (2019-2023)。

## Резюме

Многие страны с низким и средним уровнем доходов используют национальные планы в области офтальмологической помощи, чтобы направлять усилия на повышение качества офтальмологических услуг. Всемирная организация здравоохранения признает, что для формирования этих планов необходимы фактические данные. Авторы оценили фактические данные, которые были включены в выборку из 28 национальных планов офтальмологической помощи, созданных с 2013 года, когда Всемирная ассамблея здравоохранения утвердила план действий на основании резолюции «Всеобщий доступ к здоровью глаз: глобальный план действий на 2014–2019 гг.». В большинстве стран (26, 93%) были указаны оценки распространенности слепоты, и 18 стран (64%) включили в свой план целевой показатель хирургии катаракты. Другие данные редко предоставлялись или использовались для установления поддающихся количественной оценке целевых показателей. Ни одна страна не предоставила фактические данные из систематических обзоров или исследований на основе решений. Это ограниченное использование фактических данных отражает их низкую доступность, а также свидетельствует о неполном использовании существующих данных. Например, несмотря на наличие данных, дезагрегированных по половому признаку, а также данных об охвате хирургией катаракты, полученных в ходе обследований в 20 странах (71%), эти данные были представлены в планах офтальмологической помощи только девяти стран (32%). Только три страны установили показатели, дезагрегированные по половому признаку, и только одна страна установила целевой показатель для охвата хирургией катаракты для будущего мониторинга. Почти все страны признали необходимость укрепления информационной системы в сфере здравоохранения, и почти одна треть стран запланировала провести оперативные или интервенционные исследования. Необходимо определять и поддерживать реалистичные стратегии, чтобы воплотить эти намерения в действие. Чтобы получить представление о том, как страна может укрепить свой основанный на фактических данных подход к планированию в области офтальмологической помощи, мы изучаем процесс, который ведется для разработки седьмого национального плана Кении (2019–2023 гг.).

## Introduction

Accurate, reliable and timely data are required for priority setting, planning and delivering good quality health care to all. These data are necessary, but not sufficient, for countries to plan and effectively manage health programmes.^1^ The data also need to be used and this requires acknowledging their value in achieving agreed targets and outcomes.^2^ In pursuit of universal eye health, countries need to consider what data are available and the mechanisms to promote data collection, interpretation and use. This paper examines current practice, and advocates for more widespread and nuanced data from multiple sources to inform policy and practice, thus contributing not only to universal eye health, but also to promoting universal health coverage (UHC) more generally.

The World Health Assembly has guided the development of national eye-care plans for the past 15 years. The Global Initiative for the Elimination of Avoidable Blindness, *Vision 2020: the right to sight*,^3^ was launched by the World Health Organization (WHO) in 1999. In 2003, resolution WHA56.26 urged Member States to establish national eye-care plans in partnership with the WHO and in collaboration with nongovernmental organizations (NGOs) and the private sector.^4^ The process of developing a national plan provides the opportunity for a country’s stakeholders to communicate about their activities, and for the health ministry to guide coordinating mechanisms for stakeholders from different sectors and share relevant policies and priorities. In many countries, these plans have become important documents for advocacy, coordination and planning to improve eye services at the national level.

Subsequent resolutions (WHA59.25 in 2006; 62.1 in 2009; 66.4 in 2013)^5^^–^^7^ consistently recognized the importance of evidence to inform eye-care plans, specifically monitoring and evaluation data and documentation of good practices and effective models of care.^4^ Furthermore, the resolutions recognized the need to build capacity for epidemiological and health-systems research within low- and middle-income countries.^6^ *Universal eye health: a global action plan 2014–2019* was endorsed by the World Health Assembly in 2013 (resolution WHA66.4)^7^ and reaffirmed the importance of using a range of forms of evidence including epidemiological, monitoring and operational research data.^8^ The WHO and other global health advocates routinely acknowledge the importance of data to drive priority-setting, decision-making, planning, management and strategy. However, these organizations also highlight the inadequacies in quality, completeness, availability, timeliness, accessibility and use of such evidence.^2^ These limitations pose a major barrier to the use of evidence by policymakers.^9^

The United Nations’ *Transforming our world: the 2030 agenda for sustainable development*, and the corresponding sustainable development goals (SDGs)^10^ provide an opportunity to strengthen evidence for universal eye health in two main ways. The first is the recognition by WHO and other development partners that countries’ health information systems must be strengthened to generate the information needed for decision-making and for tracking progress towards the SDG targets.^11^^–^^13^ The second is the specific focus of the SDGs on leaving no one behind, by ensuring services reach those people previously most neglected.

In this paper, we discuss the main sources of evidence that can inform eye-care plans and reflect on their incorporation in current national universal eye health plans. We then describe the evidence-informed approach Kenya is currently taking in the development of its seventh national eye-care plan (2019–2023) to share insights that may assist development of national eye health planning and strategy more broadly.

## Use of evidence

### National universal eye health plans

To explore the use of evidence in universal eye health plans in low- and middle-income countries we assembled a sample of 28 national plans developed since the World Health Assembly endorsed resolution WHA66.4 in 2013 (Box 1). These plans were obtained by contacting 88 traceable national eye-care coordinators, five global and regional WHO eye health staff, six global and regional International Agency for the Prevention of Blindness staff, 11 NGOs and 22 key experts in the field. Contact was made between May 2017 and June 2018. Reasons provided by 51 countries unable to provide a plan included: the previously expired plan had not been replaced; eye-care planning was fully integrated into noncommunicable diseases or other general health plans; plans were still being developed; or plans were waiting for health ministry endorsement.

Box 1 **Examples of national eye-care plans generated after the World Health Assembly Resolution on universal eye health, May 2013**

**African Region**

Botswana, 2015–2019; Burkina Faso, 2016–2020; Cameroon, 2015–2019; Ethiopia, 2016–2020;^a^ Mozambique, 2015–2019; Nigeria, 2015–2020;^a^ Togo, 2015–2019; Uganda, 2016–2020; Zambia, 2017–2021.

**Region of the Americas**

Belize, 2015–2020; Bolivia (Plurinational State of), 2017–2021; Colombia, 2016–2022; El Salvador, 2014–2019; Honduras, 2015–2019; Mexico, 2014–2019; Peru, 2014–2020; Venezuela (Bolivarian Republic of), 2014–2019.

**Eastern Mediterranean Region**

Afghanistan, 2017–2021; Egypt, 2014–2019; Libya, 2014–2019; Morocco, 2014–2019; Pakistan, 2015–2019; Yemen, 2017–2020.

**South-East Asia Region**

Indonesia, 2017–2030; Myanmar, 2017–2021.

**Western Pacific Region**

Cambodia, 2016–2020; China, 2016–2020; Papua New Guinea, 2018–2021.^a^

^a^ Draft awaiting sign-off from health ministry.Note: Plans were completed after World Health Assembly resolution 66.4, *Towards universal eye health: a global action plan 2014–2019*.^7^

We included only plans that were focused on eye care and excluded general health plans with eye care as a component. We also only included plans that mentioned WHA66.4^7^ or the *Universal eye health: a global action plan 2014–2019*.^8^ The resulting sample (Box 1) is therefore a subset of all existing plans in low- and middle-income countries and represents those countries willing and able to share a current plan.

### Monitoring of priority indicators

Of the universal eye health priority indicators (Table 1), most national eye-care plans reported baseline information on the prevalence (26 countries, 93%) and causes (25 countries, 89%) of blindness, followed by cataract surgical rate and number of ophthalmologists (23 countries, 82%, for both indicators). Cataract surgical coverage was the indicator least often reported (by only nine countries, 32%), despite being generated by the Rapid Assessment of Avoidable Blindness methods used by 20 countries to report blindness prevalence estimates. This suggests that reasons other than availability contribute to the underuse of data on cataract surgical coverage in eye-care plans.

**Table 1:** Reporting of the priority indicators from the *Universal eye health: a global action plan 2014–2019* in a sample of 28 national eye-care plans from low- and middle-income countries

Universal eye health priority indicato**r^a^**	Notes	Anticipated source	No. (%) of plans		
			Quantifying current eye health situation	Citing sources of evidence	Reporting future measurable objective or target
Prevalence of blindness	Prevalence of visual acuity < 3/60, preferably disaggregated by age and sex	Population-based survey	26 (93)	25 (89)	11 (39)
Prevalence of visual impairment	Prevalence of visual acuity < 6/18 ≥ 3/60, preferably disaggregated by age and sex	Population-based survey	14 (50)	14 (50)	2 (7)
Causes of blindness	Causes of visual acuity < 3/60, preferably disaggregated by age and sex	Population-based survey	25 (89)	23 (82)	2 (7)
Causes of visual impairment	Causes of visual acuity < 6/18 ≤ 3/60, preferably disaggregated by age and sex	Population-based survey	11 (39)	11 (39)	NR
Cataract surgical rate	Number of surgeries performed per year, per million population	Health information system	23 (82)	7 (25)	18 (64)
Cataract surgical coverage	Proportion of individuals with bilateral cataract causing visual impairment who have received cataract surgery on one or both eyes, preferably disaggregated by age, sex, place of residence (urban/rural) and district	Population-based survey	9 (32)	6 (21)	1 (4)
Quantity of ophthalmologists	Number of medical doctors certified as ophthalmologists by national institutions based on government-approved certification criteria	Professional register	23 (82)	8 (29)	14 (50)
Quantity of optometrists	Number of optometrists certified by national institutions based on government-approved certification criteria	Professional register	20 (71)	7 (25)	11 (39)
Quantity of allied ophthalmic personnel	Numbers of allied ophthalmic personnel comprising professional categories, which need to be specified by a reporting Member State	Administrative records: government, nongovernmental, private sector	18 (64)	4 (14)	13 (46)

NR: not reported.

^a^ From the *Universal eye health: a global action plan 2014–2019.*^8^

Notes: Included countries: Afghanistan, Belize, Bolivia (Plurinational State of), Botswana, Burkina Faso, Cambodia, Cameroon, China, Colombia, Egypt, El Salvador, Ethiopia, Honduras, Indonesia, Libya, Mexico, Morocco, Mozambique, Myanmar, Nigeria, Pakistan, Papua New Guinea, Peru, Togo, Uganda, Venezuela (Bolivarian State of), Yemen, Zambia.

Few countries used baseline data to construct any measurable targets, apart from the cataract surgical rate; almost two-thirds (18 countries, 64%) set a target cataract surgical rate (Table 1). This general lack of measurable targets limits a country’s ability to monitor progress or to evaluate the implementation of the eye-care plan, and may reflect concerns regarding the lack of available data. For example, none of the included countries had data from two national blindness surveys to permit detection of a change in blindness prevalence over time at the national level.

Monitoring of inequalities in eye care needs to be strengthened. The universal eye health plan calls for prevalence and cataract surgical data to be disaggregated by age, sex and place of residence.^8^ Almost all eye health surveys report blindness and visual impairment prevalence disaggregated by sex^14^ and the disparity between women and men has been documented for almost two decades.^15^ However, only nine countries (32%) reported a baseline prevalence indicator disaggregated by sex, and only three specified the intention to disaggregate an indicator in the future: Mexico and Myanmar by age and sex; and Zambia by sex, urban/rural area and disability. To ensure we leave no one behind, the reasons why countries do not use available disaggregated data in policies and plans need to be explored and solutions identified.

### Mains sources of evidence

The universal eye health plan anticipated that the main sources of evidence to report priority indicators would be population-based surveys, government health information systems and administrative data (Table 1).^8^ We discuss the use of each of the sources in existing plans here.

#### Population-based surveys

**Fig. 1:**
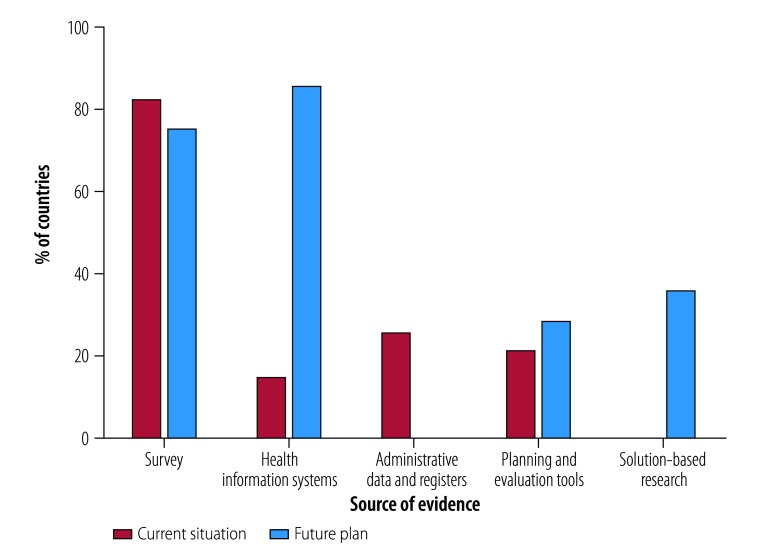
Sources of evidence in national eye-care plans from low- and middle-income countries

Notes: We analysed a sample of 28 national eye-care plans generated since *Universal eye health: a global action plan 2014–2019* was endorsed by the World Health Assembly in 2013.^8^We noted whether evidence was cited to describe the current situation and was planned for use in the future. Solution-based research includes operational, implementation and health-systems research. Included countries: Afghanistan, Belize, Bolivia (Plurinational State of), Botswana, Burkina Faso, Cambodia, Cameroon, China, Colombia, Egypt, El Salvador, Ethiopia, Honduras, Indonesia, Libya, Mexico, Morocco, Mozambique, Myanmar, Nigeria, Pakistan, Papua New Guinea, Peru, Togo, Uganda, Venezuela (Bolivarian State of), Yemen, Zambia.

Population-based surveys were the most commonly cited source of evidence in plans (23 countries, 82%), primarily reporting prevalence and causes of blindness and, to a lesser extent, cataract surgical coverage. Similarly, most countries (21, 75%) stated their intention to undertake a prevalence survey as one of the activities in their plan (Fig. 1).

The number of surveys undertaken to measure blindness and vision impairment has increased in the past two decades,^16^ largely due to the development of the Rapid Assessment of Avoidable Blindness method^17^ which was the source of data cited by 20 of the 23 countries citing survey data. The method is quicker and easier than full population surveys and produces estimates that correlate well with full population surveys.^18^ Rapid Assessment of Avoidable Blindness routinely reports outcomes disaggregated by age and sex, and trials are currently underway to expand the social variables collected to enable monitoring of more dimensions of disparity.^19^

Some limitations of surveys for national planning are the lack of frequency in conducting them and that most are conducted at the subnational level. A recent call has been made for visual acuity assessment to be added to UHC monitoring tools such as district health surveys;^20^ if implemented, this would provide regular national-level data on blindness and visual impairment. Until this is a reality, data from rapid assessments and other surveys at the subnational level will continue to be the most commonly available survey data for eye-care planning.

#### Health information systems

The 23 countries (82%) reporting data on the cataract surgical rate rarely cited the source of the information, and only six specified whether private-sector data were included alongside information from the public health sector. Seven countries (25%) integrated eye health monitoring with health ministry systems and a further 14 (50%) indicated a need for this to occur. Furthermore, almost all countries recognized the need to strengthen their health information systems to support monitoring of eye-care services and policy (24 countries, 85%; Fig. 1). This integration and strengthening would provide real-time indicators of service use, repeated observations over time and data from all participating health facilities throughout a country.^21^

However, to realize the full potential of eye health information systems, weaknesses in relation to data completeness and accuracy will need to be addressed.^1^ Eye health monitoring will benefit from interventions that ensure staff working in eye departments are engaged in the monitoring process, understand its value and receive training, feedback and supervision.^22^^–^^24^

#### Administrative data

Accurate and up-to-date health workforce data enable countries to plan more equitable and effective distribution of relevant workers and to make future projections.^25^ While countries with low numbers of relevant staff can easily monitor eye-care personnel, in countries with more complex systems of health-care delivery the need for data external to the health ministry may make data collection challenging.^25^^,^^26^ Health workforce data were commonly reported in national eye-care plans, but the source was cited by only eight countries (29%) and six (21%) specified whether or not private practitioners were included. None of the country plans specified an intention to strengthen data on the eye health workforce (Fig. 1).

### Other sources of evidence

While the data sources mentioned above were the most frequently cited in the 28 national eye health plans reviewed, other sources can also be mobilized to assist planning and monitoring.

#### Planning and evaluation tools

Decision-makers can use evaluations of existing health plans to identify implementation issues and to produce a situation analysis on which to base subsequent plans.^27^ Most countries (25, 89%) referred to using a situational analysis to inform the planning process, but only six (21%) described how this occurred, for example, by using strengths, weaknesses opportunities, threats analysis or the eye care service assessment tool.^28^ Looking ahead, eight countries (29%) listed the intention to evaluate implementation of the plan (Fig. 1). Two planning tools recently released by WHO can strengthen the planning and evaluation process by systematically documenting eye care^28^ and diabetic retinopathy services.^29^ The Rapid Assessment of Avoidable Blindness Planning module currently under development^19^ may also help bridge the evidence–policy gap.

#### Solution-based research

When developing national plans, decision-makers ideally draw on good quality, timely evidence (e.g. systematic reviews and intervention, implementation, operational and health systems research) that describes what works, for whom and in what circumstances. Unfortunately, little of this evidence exists for eye health in low- and middle-income countries.^30^^–^^32^ Indeed, none of the countries cited a systematic review or any solution-based research to justify a policy approach or decision in their national plan (Fig. 1). However, 10 countries (36%) listed the intention to conduct solution-based research within their plan. In addition, 12 countries (43%) recognized the need to strengthen the research process, including by establishing a research agenda, building research capacity and improving the use (or translation) of research in policy and practice. These intentions provide an opportunity to explore promising strategies and identify factors that influence service provision^33^^–^^37^ in different settings and to subsequently evaluate the use of such evidence. Eye health research in low- and middle-income countries is likely to remain under-resourced, so it is essential that development partners, funders and researchers collaborate innovatively with countries to identify, generate and disseminate the most relevant evidence.^32^^,^^38^

#### Global estimates

Recent years have seen increased investment in global health metrics and the development of synthesis and modelling methods. While global estimates play an important role in setting global priorities, they are of limited value in planning at the national level.^39^ The investment in deriving global estimates ought to be balanced with building capacity within countries to collect, analyse, interpret and use data for national and subnational planning.^39^^,^^40^

#### Mobile device applications

Researchers are currently testing several mobile device applications for eye care that may provide useful information for policy and planning. Two notable examples are the BOOST application (Better Operative Outcomes Software Technology) for monitoring outcomes of cataract surgery^41^ and the Peek application (Portable Eye Examination Kit) for vision screening and referral.^42^ Any scale-up of these tools needs to be evaluated in terms of their acceptability, feasibility and cost of widespread use in eye health systems, including the potential for integration within existing national eye health information systems.

## An example from Kenya

Here we draw on the broader findings of existing plans outlined above to reflect on how countries can strengthen the use of evidence in eye-care planning. Kenya is used as a case study, as the current strategic plan for eye health and blindness prevention (2012–2018) is ending and the country has begun to develop its seventh eye-care plan (2019–2023).

As in other countries, eye health needs and services in Kenya compete with many other priorities. However, eye health receives government support at the national level and Kenya’s eye-care plans are annexed to the national health sector strategic plan. The ophthalmic services unit at the health ministry develops annual operational plans and budgets based on the national eye-care plan. These identify the activities covered by health ministry funding and the activities for which external support is required.

### Sources of evidence in Kenya

The next eye-care plan in Kenya can draw on a broad range of evidence sources, including reports not published in the scientific literature (Box 2). National level survey data are not available and there are no current plans to conduct a national survey of the prevalence of blindness and visual impairment. This means that the ability to monitor prevalence and coverage indicators at the national level will continue to be limited. In the forthcoming plan, rather than excluding targets that have no guaranteed way to be measured, the global priority indicators will be included with an explicit statement that they will only be measured should appropriate surveys be undertaken. Alongside these targets, the plan will provide a list of priority counties (districts) for future surveys to help direct support from donors, researchers and development partners should funds for surveys become available.

Box 2 **Potential sources of evidence for Kenya’s next eye-care plan**

**Surveys (all ages)**

Surveys in eight regions, 1990: Baringo, Kajiado, Kakamega, Kisii, Kwale, Meru, Nyanza, Nyeri.^43^

Trachoma surveys: baseline and impact surveys from all counties, 2004–2017.

**Surveys (adults)**

Rapid Assessment of Avoidable Blindness survey: Nakuru, 2004; Kericho, 2007; Embu, 2007; Homa Bay, 2010; Kwale, 2011; Embu (Mbeere), 2013.

Other blindness prevalence surveys: Nairobi, 2002; Nakuru, 2007/2008.

Cohort studies (incidence): Nakuru, 2013/2014.

**Health information systems**

Eye facility monthly reports within the national District Health Information System 2 data platform (2012–2017).

Indicators include: number of new and returning patients; number of admissions; clinical diagnosis disaggregated by age (< 5, 5–15 and ≥ 16 years), sex and visual status (not vision impaired, moderate and severely visually impaired and blind); and surgeries disaggregated by surgery type, age group (as above) and sex.

Completeness, accuracy and timeliness of these data are all concerns and a data quality review of the eye health information systems will be completed in 2018 to identify appropriate quality improvement interventions to implement and evaluate.

A feasible and acceptable measure of cataract surgical quality will be trialled at the facility level, possibly using the BOOST (better operative outcomes software technology) application.^41^

Inequality monitoring in eye departments will be trialled in 2018 to determine the feasibility of expanding the social variables collected (e.g. socioeconomic status, place of residence, disability and social support).

**Administrative data**

Human resources: Medical Board; Nairobi University; ophthalmic clinical officer register; College of Ophthalmology of Eastern Central and Southern Africa; Nurses Council register; health ministry ophthalmic services unit records.

Equipment and consumables: audit of eye departments every 2 years.

**Planning and evaluation tools**

Evaluation report: implementation of current eye health plan, 2012–2018.

Eye care service assessment tool, 2017.^28^

Eye health system assessment, 2015.^44^

**Guidelines**

Completed: retinoblastoma,^45^ diabetic retinopathy.^46^

Forthcoming: retinopathy of prematurity, glaucoma.

**Solution-based research**

Completed: school vision screening and referral.^42^

Forthcoming: community screening and referral; diabetic retinopathy community and practitioner behaviour change; evaluation of trachoma strategy.

**Other**

Reports from some mission hospitals, nongovernmental organizations, private hospitals.

- Cataract surgical audits (e.g. postoperative outcomes) from six eye departments.
- Diabetic retinopathy service use at Kenyatta national hospital.
- Kenya trachoma situational analysis report, 2013.
- Systematic reviews on relevant topics.

A priority in the plan will be to strengthen the eye health information systems and the capacity to evaluate policies at the facility, subnational and national levels using routinely generated data in the health information systems (Box 2).^21^^,^^32^ Other sources of evidence that will be used in the next plan include administrative data; information collected using the recent eye care service assessment tool^28^ and eye health system assessment approach;^44^ clinical guidelines; and solution-based research including studies assessing how to improve vision screening and referral,^42^ and trachoma and diabetic retinopathy services.

A challenge Kenya shares with many countries is the incomplete provision of data from the private sector (currently around 30 inpatient facilities). Increasing the information provided by private providers is another area of focus of the next plan. Private practitioners are invited to participate in the planning process and to nominate a representative on the national coordinating committee. In the next plan, the ophthalmic services unit will compile a list of private facilities as an annex. The unit will prepare an outline of the planning process and explain the value of generating and using data from all sectors. This outline will be shared with all private facilities along with a request to provide data in a standard format.

### Leaving no one behind

Kenya has committed to implementing the 2030 agenda for sustainable development and accordingly Kenya’s next eye-care plan will have a greater focus on equity. A trial is underway to determine the feasibility of expanding the social variables collected in the eye health information systems beyond age and sex (Box 2). Any disparities in eye health experienced by disadvantaged population subgroups will be used to set disaggregated targets (e.g. socioeconomic status, urban/rural, disability and social support) for ongoing monitoring.

**Fig. 2:**
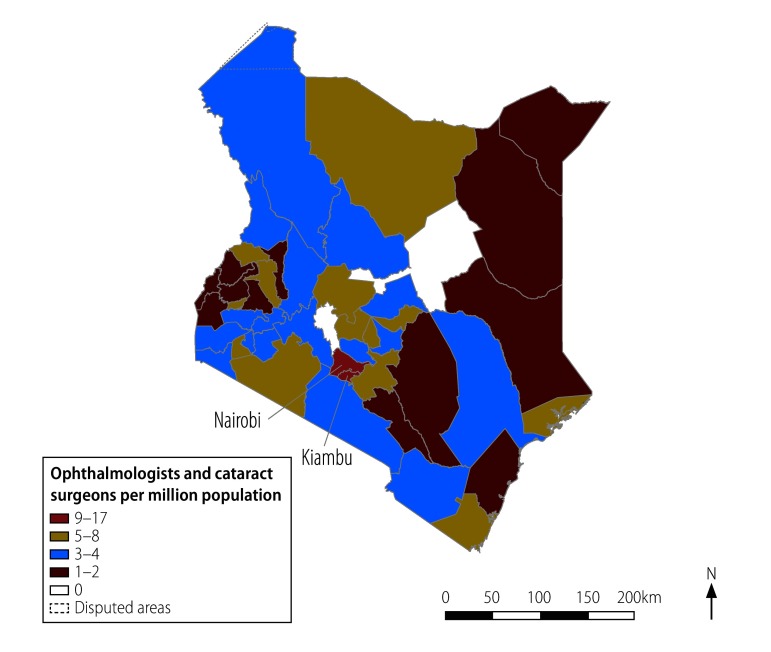
Distribution of public sector ophthalmologists and cataract surgeons across the 47 counties of Kenya, December 2017

Source: The population of Kenya was 49.55 million in 2017, projected from the 2009 census by the United Nations Children’s Fund.^47^ Data are the distribution of 115 ophthalmologists and 121 cataract surgeons (collectively 236 surgeons) from the ophthalmic services unit, Ministry of Health, Kenya.

In addition, subnational (intercounty) inequality of health system inputs and service outputs will be monitored to help target policies towards the counties most in need. For example, a map helps to highlights the high density of surgeons in the urban counties of Nairobi and Kiambu compared with rural counties with low or no surgeons (Fig. 2). Other intercounty monitoring in future will include stockouts of surgical consumables, cataract surgical rate and the proportion of cataract surgeries covered by health insurance.

### Strengthening the use of evidence

In addition to having more evidence to draw on when developing the next eye-care plan (Box 2), the eye health research workforce has also increased, with four Kenyan ophthalmologists recently completing postgraduate research degrees exploring policy-relevant clinical and service delivery questions. Furthermore, the planning process will also be enhanced. As in the past, the next plan will be based on a situation analysis, a review of the current plan and a SWOT (strengths, weaknesses opportunities, threats) analysis. In addition, a monitoring, evaluation and review framework will be developed to guide the situation analysis and to monitor implementation of the subsequent plan.^27^ Once this information is collated, the health ministry will host a summit of policy-makers, service providers, training institutions, NGOs, WHO Country Office Kenya, researchers and development partners. The summit will enable participants to discuss the relevant evidence from the health information systems, and epidemiological, intervention, operational and implementation research. Feedback from this summit will be incorporated into the subsequent plan.

The monitoring and evaluation framework for Kenya set out in Box 3 will contain the key attributes for monitoring national plans outlined by WHO.^48^ Kenya’s eye-care plans have previously included activities to strengthen monitoring and will continue to do so, although in a more explicit way. For example, a research agenda that specifies priority research areas will also be an annex to the eye-care plan to embed evidence into the policy process.

Box 3 **Key attributes of the monitoring and evaluation framework Kenya’s next eye-care plan**

- Incorporate data into indicators by setting SMART (specific, measurable, attainable, relevant and timely) targets.
- Specify data sources and gaps and outline data collection and information flow (e.g. prevalence of blindness and cataract surgical coverage can only be monitored if further surveys are conducted).
- Describe data completeness and accuracy (e.g. the extent to which the private sector was invited to provide data and the extent to which it complied).
- Take steps to improve data quality (e.g. data quality review of the eye health information systems).
- Strengthen the capacity of the eye health workforce in monitoring.
- Build consensus between producers and users of data.
- Prospectively plan, implement and disseminate an evaluation.

Note: Based on World Health Organization guidelines on monitoring, evaluation and review of national health strategies.^48^

## Conclusion

When generating evidence for eye-care plans, countries, researchers, and funders have given priority to undertaking epidemiological studies and the past two decades have seen an increase in the number of countries with data from population-based surveys. Unfortunately, the use of evidence from these and other sources to inform eye health plans is currently limited. Countries commonly recognize that improving eye health planning and monitoring will depend on enhanced health information systems, thus linking eye health to broader improvements in health systems and health management information systems. Production of solution-based research in eye health is currently so limited it can barely influence policies. Innovative and collaborative country-led strategies are required to identify, generate, disseminate and use the most relevant evidence for universal eye health. 

Consideration of equity is currently weak in eye health plans. The SDGs help reinforce the need for more nuanced and disaggregated data that will help shape priorities and address the needs of the most marginalized people. A wide range of data sources can be used that need to go beyond the minimal data currently collected in many settings. Furthermore, WHO could provide more technical guidance to countries on practical ways to incorporate equity into their eye-care plans.

Kenya provides valuable insights into what can be done at country level to improve data collection and use. We argue that promoting universal eye health is central to achieving UHC and that countries and their development partners should work collectively to advocate for and achieve improved outcomes for largely preventable and treatable conditions.
